# AVOKE: an open-source toolbox for audiovisual web experiments in jsPsych

**DOI:** 10.7717/peerj.20544

**Published:** 2026-01-07

**Authors:** Shreshth Saxena, Jackson Shi, Lauren Fink

**Affiliations:** Department of Psychology, Neuroscience, and Behaviour, McMaster University, Hamilton, ON, Canada

**Keywords:** Online, Web-based, Studies, Inclusivity, Remote, Javascript, Webcam, Youtube, Animation, Calibration

## Abstract

As web-based experiments become increasingly popular, the need for advanced, accessible research methods is greater than ever. Existing solutions for designing and building an experiment are often closed source and proprietary, which limits configurability, affordability, and testability. Moreover, inconsistent terminologies, domain-specific coding and documentation, and practices such as open-washing impair the ease of adoption and effective use of these tools by novice researchers and practitioners. To address these shortcomings, we release AVOKE—a collection of free and open source tools for web-based behavioural experiments. AVOKE extends the functionalities of the widely adopted jsPsych library, matching community standards for code reusability and comprehensive documentation. The current release of AVOKE supports temporally-precise presentation of audiovisual media (including generated animations, preloaded files, and external sources like YouTube), as well as the collection of behavioural responses, like keypresses and video capture (*e.g.*, for recording face videos of participants). This paper elaborates on the functionalities, implementation, and usage of the included plugins and extensions in AVOKE. These extensions and plugins have been validated with simulation testing and utilised in previous and ongoing studies. AVOKE is openly available at https://www.github.com/beatlab-mcmaster/AVOKE and archived on Zenodo. We welcome contributions to AVOKE and discuss potential future additions to ease the development of advanced web-based experiments for all users.

## Introduction

Web-based experiments emerged soon after the invention of the World-Wide-Web in 1992, gaining momentum with the spread of web-browsers and internet-based forms that enabled sending back responses from distributed clients to a central server. Since then their popularity has been on a consistent rise, often seeing dramatic surges during critical times, such as the COVID-19 pandemic. The proliferation of web-based experimentation can be attributed to its many advantages. For example, web experiments enable greater accessibility, allowing participation from individuals across diverse socio-cultural backgrounds, including those with unique or less common traits, such as social anxiety or immunocompromisation (see Reips (2000) for a discussion). By allowing access over internet, web experiments can bypass geographic, logistical, linguistic, and economic constraints far more effectively than laboratory based studies. They offer virtually-uncapped parallel participation that is often more economical in terms of cost, time, space, and effort, compared to traditional laboratory research (Hartshorne et al., 2019). Consent and voluntary participation is also better administered by eliminating the need for experimenter supervision and synchronous experimenter-participant interactions. Further, the external validity of findings is boosted when experiments can be conducted outside laboratory environments and, because the experiment materials and protocols can be shared openly and accessed by other researchers for verification or adaptation, replicability and reusability are also enhanced (Reinecke & Gajos, 2015).

In addition to being well-suited for online studies over the internet, web-based experiments have also been recognised as a best practice for designing laboratory studies with in-person participants, and, naturally, for hybrid studies that recruit both in-person and remote participants. This practice is grounded in the fundamental asymmetry of accessibility: while any web-based experiment can easily run in a lab, a stand-alone laboratory experiment is not always compatible for online deployment (Reips, 2002). Conducting a laboratory study with web-based experimentation preserves the benefits of re-using well-established and standardised methods, alleviating experimenter biases with unsupervised protocols, and guaranteeing future replicability and scalability of current methods.

**Figure 1:**
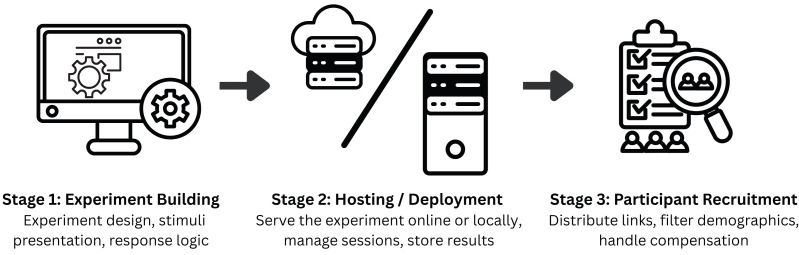
Web experimentation workflow. AVOKE contributes to the experiment building stage of this workflow emphasizing ease of use for new users, code reusability, and advanced configurability through an open-source implementation. Experimenter’s careful selection of hosting and participant recruitment tools further supports secure data handling and effective dissemination of studies.

With the increase in popularity, several tools for developing web-based experiments have emerged. These tools are developed to assist with the three primary stages of the web-based experimentation workflow, as illustrated in Fig. 1. Experiment building (Stage 1) is the core of this workflow where the experimenter needs to implement all experiment logic and materials to run on a web-browser. Once the experiment is developed, it must be served (Stage 2) for the participants (clients) to access it. The experiment can be served either locally–such as in laboratory-based, in-person studies–or on the cloud–for remote participants to access it over the internet. Finally, in Stage 3, the experiment is securely shared with a target pool of participants using tools that facilitate participant recruitment. Stages 2 and 3 are not always mandatory. For instance, if an experimenter chooses to develop the experiment on the same computer that the participants will access it from, Stage 2 can be omitted. Similarly, when distributing an experiment’s link through personal invites, a dedicated participant recruitment (Stage 3) tool is not required. These stages are nonetheless important for robust data management and privacy practices (Stage 2) as well as transparent, ethical, and scalable participant engagement (Stage 3). Table 1 provides an overview of existing tools and their functionalities across these workflow stages. In this paper, our focus lies primarily on Stage 1—experiment building that underpins replicability, openness, and extensibility of experimental procedures.

**Table 1:** An overview of representative web-based experiment tools.

**Tools**	**Functionality**	**Interfacing**	**Availability**
jsPsych (de Leeuw, Gilbert & Luchterhandt, 2023), Shiny (Chang et al., 2025), Psychtoolbox (Kleiner, Brainard & Pelli, 2007)	Experiment building	Code/script based	Free & Open Source
OpenSesame (Mathôt, Schreij & Theeuwes, 2012), lab.js (Henninger et al., 2021), PsychoPy/JS (Peirce et al., 2019)	Experiment building	GUI-based with optional scripting	Free & Open Source
JATOS (Lange, Kühn & Filevich, 2015)	Hosting / deployment	GUI + code-based	Free & Open Source
OpenLab (Shevchenko, 2022), Pavlovia (Open Science Tools, 2025)	Hosting / deployment	Web-based GUI interface	Free (limited) & Paid tiers
Prolific (Palan & Schitter, 2018), Sona (Sona Systems Ltd., 2025)	Participant recruitment	Web-based GUI interface	Closed Source (commercial)
Gorilla (Anwyl-Irvine et al., 2020), LabVanced (Finger et al., 2017)	Experiment building, hosting/deployment, and participant recruitment (limited)	Web-based visual builder (GUI) with sandboxed code blocks	Closed Source (commercial)

Most experiment building tools use native web technologies, such as HTML, CSS, JavaScript, and PHP (de Leeuw, Gilbert & Luchterhandt, 2023; Henninger et al., 2021) for developing and hosting web-based experiments. Alternatives that allow building web experiments with other popular languages, such as Python, R, and MATLAB, also exist (Peirce et al., 2019; Kleiner, Brainard & Pelli, 2007; Chang et al., 2025; Estephan, 2022). These tools automatically export or transpile the experiment logic into native web technologies to run on participants’ browsers. To further simplify the process for researchers, many experiment builders have incorporated Graphical User Interface (GUI) components and human-readable coding (*e.g.*, through JSON objects) to develop experimental procedures, lowering the barrier to entry for novice users and non-programmers (Mathôt, Schreij & Theeuwes, 2012; Henninger et al., 2021). Finally, this trend has also been joined by for-profit, commercial companies that offer monetised solutions to build and host experiments, focussing on ease of use, responsive customer support, and, in some cases, participant recruitment services (Finger et al., 2017; Open Science Tools, 2025; Anwyl-Irvine et al., 2020). A non-exhaustive overview of the existing tools available for web-based experiment development is provided in Table 1.

While each of these existing tools overcome some of the shortcomings commonly known to impede the adoption of web-based experimentation, they also present certain constraints, which makes it complicated for an experimenter looking to make the right choice (Sochat et al., 2016). Of the existing tools for building web-based experiments (see Table 1), code-based libraries and packages have been around for the longest and offer great customisation possibilities for novel and complex paradigms. The adoption and re-usability of these libraries is, however, subject to the availability of comprehensive documentation, open-sourced code implementations, and reproducible testing suites. The GUI or Domain Specific Language (DSL)-based experiment builders offer a quick and easy-to-use solution that requires little to no coding experience. However, these confine the experimenter to a limited set of pre-designed objects and routines (*e.g.*, stimuli, responses, conditions, loops), which inherently restricts support for unconventional stimulus-media types (*e.g.*, livestreams, 360° video) and complex experimental timelines (*e.g.*, dynamic, adaptive, non-linear designs). To circumvent this issue of limited customisation, these tools often allow the inclusion of custom coding blocks; however, the custom code needs to be sandboxed for security and compatibility within the platform’s ecosystem. As a result, configurability remains restricted: users still lack direct access to native browser application programming interfaces (APIs), and in extreme cases, extensive custom coding can overwhelm the experiment design, compromising performance and negating the benefits of GUI-based development. Moreover, the platform-specific concepts/terminologies in these experiment builders are often not transferable outside the ecosystem, which builds a dependency on limited specialised expertise and can pose challenges for new users seeking support. Proprietary, closed-sourced solutions from commercial vendors further reduce this reusability and customisation while also impacting affordability for larger studies, reproducibility of findings, and validation of experimental paradigms by external parties. Standardisation of experimental tools and paradigms is, therefore, critical for the widespread adoption, vetting, and reproducibility of web-based experiments (Reinecke & Gajos, 2015).

### The jsPsych framework

The goal of standardisation in web-based experiment building is best realized with open-source, publicly available, and actively maintained tools that offer both high customisability to add advanced methods in web-based experiments and comprehensive documentation to increase accessibility for new users. Commercial and simplified experiment builders, while often user-friendly, fundamentally lack the flexibility and transparency to realize this goal. We, therefore, turn to open-source experiment building libraries developed with native web technologies such as jsPsych. jsPsych (de Leeuw, Gilbert & Luchterhandt, 2023) is a community-driven, research-grade JavaScript library that (i) allows quick-and-easy experiment building through abstracted modules that make it straightforward to design survey forms and simple experiment paradigms without requiring prior web development experience, (ii) provides good documentation and code customisation that allows integration of advanced methods and complex paradigms that were previously only possible in laboratory experiments, (iii) supports simulating participant behaviour for robust testing of the developed experiment, and (iv) offers clear guidelines and a dedicated open source repository for community contributions to facilitate sharing and re-using work from external contributors.

JsPsych provides fine-grained control over stimulus presentation and response collection with the help of two design concepts–plugins and extensions. Plugins serve as the building blocks for every experiment created in jsPsych. They abstract the core functionalities of an experimental trial and allow the experimenter to create custom experiments without having to write all the underlying code from scratch. On the other hand, jsPsych extensions are meant to be additions to any plugin to create extra features without interfering with the plugin itself. These extensions typically do not provide direct on-screen elements for participant interaction and are therefore used in conjunction with plugins, executing tasks in the background. In this context, the term extension is appropriate, as they serve to augment and extend the functionality of existing plugins. The modular architecture, decentralised development, active community, and proven track record in peer-reviewed behavioural research (*e.g.*, Mok et al., 2024; Strittmatter et al., 2024) make jsPsych an ideal choice for standardising practices in web-based experiment building. At the heart of this practice is the availability of a large, open collection of experimental tools and paradigms that can be easily replicated, evaluated, and upgraded *via* community contributions.

## The AVOKE Toolbox

We contribute to the existing collection of open source jsPsych plugins and extensions with AVOKE—a curated collection of tools that facilitate advanced audiovisual experimental paradigms. At the time of release, AVOKE consists of five plugins and one Extension; we will continue to develop and integrate more in future releases. AVOKE is released with easy-to-follow templates and examples, along with comprehensive documentation to lower barriers in required expertise, enabling researchers unfamiliar with coding to begin building their own web-based experiments. The released paradigms easily integrate with any jsPsych study and can be customised with the help of provided parameters. AVOKE also emphasises future usability and customisation of proposed paradigms with active maintenance and contribution guidelines. Below, we describe each of the component plugins and the extension included in AVOKE. Documentation and examples of code usage for each component plugin/extension are available in the AVOKE GitHub repository (Shi, Saxena & Fink, 2025); see the Code Availability section below.

### Video capture extension & setup plugin

Recording a participant’s video feed as they complete an experimental procedure is a common practice in laboratory-based experimentation. Beyond being useful for data quality checks and archival purposes, these recordings serve as a way to monitor participant activities, analyse their physiology and behaviour using manual or automated methods, and manipulate the experimental procedure based on real-time physiological readings or implicit behavioural measures. However, there remains a notable lack of easy-to-integrate tools that enable video recording for web-based experiments. This gap is partly due to the heightened challenge of ensuring compatibility across the diverse, open-ended range of hardware that can be used to run web-based experiments, as well as the inherently higher noise of data when web-based studies are deployed remotely. Yet, this landscape is rapidly changing. Modern web browsers now support direct and secure access to local peripheral devices without the need for specialised drivers. Furthermore, recent advances have demonstrated the feasibility of extracting robust behavioural and physiological signals—such as eye movements, motion, heart rate, and skin colour changes—from webcam recordings made in unconstrained settings (Saxena, Fink & Lange, 2024; Lugaresi et al., 2019; Boccignone et al., 2025; Sen, Bala & Gouthaman, 2023).

AVOKE provides this functionality with the video capture extension. The extension supports video recording by using connected video input devices on a user’s computer. It also supports additional features like local download, server-side storage, choosing video resolution, and splitting recorded video into smaller chunks. These features are not only essential for testing and debugging during experiment development but also for optimizing data collection for long duration studies where data size and network bandwidth are common performance bottlenecks. Video capture extension can be added to any experimental trial to record webcam video in the background. Videos are recorded in the widely supported MP4 format, facilitating efficient post-processing and analysis. Video meta data, such as filenames and recording start/stop timestamps, are added to the corresponding trial’s data.

**Figure 2:**
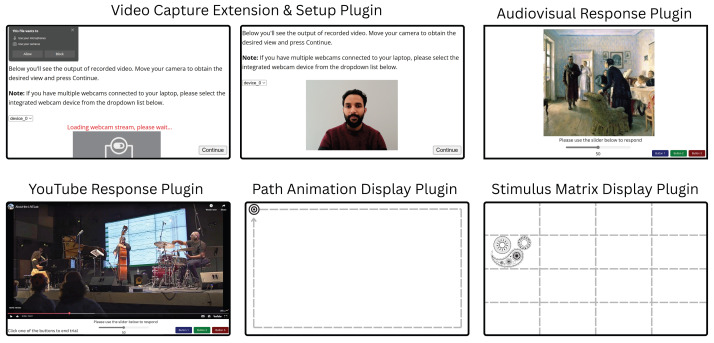
Illustrations of an example trial of each plugin included in the AVOKE toolbox. **Video capture extension & setup plugin:** The user is prompted to provide webcam permissions (top left panel), then their image is displayed (top centre panel). This feedback shows participants what will be recorded and allows them to make adjustments to suit experimental requirements (*e.g.*, move away from a background light source; remove glasses, *etc.*). **Audiovisual response plugin:** Experimenters can combine images with audio. Here, for example, Unexpected visitors by Ilya Efimovich Repin (public domain) is shown; audio not pictured. A variety of response options (*e.g.*, slider, buttons), are available. **YouTube response plugin:** Allows presentation of pre-recorded or livestreamed YouTube content, with a variety of controls and relevant output data related to timing, video resolution, participant responses, *etc.* Here we show an example video from @mcmasterlivelab’s YouTube channel; audio not pictured. **Path animation display plugin:** Animates a visual target on a 2D path. Here the smooth-pursuit calibration target from Saxena, Lange & Fink (2022) is shown. **Stimulus matrix display plugin:** Enables subdivision of the screen into a matrix of arbitrary size and presentation of any type of visual target (*e.g.*, face, pattern, *etc.*) within a specific cell. An example visual target from Saxena, Fink & Lange (2024) is shown. Note that the dashed, gray guidelines in the last two plugins are only for illustration purposes; these are not visible to the user.

Video capture extension does not allow direct user interaction during execution, which is what the video capture setup plugin is for. This plugin collects explicit permissions for camera and microphone usage from the user and allows them to select one of the available video capture devices attached to their computer for collecting data. Once the input capture device is initialised, the plugin presents a live preview of the incoming stream from that device, see Fig. 2. The video capture setup plugin also allows researchers to display any instructions to the participant before they proceed with the recording. These instructions can be presented with common HTML formatting. Using the video capture setup plugin alongside the video capture extension allows the user to clearly see what will be recorded and correct their positioning in the recording stream before proceeding.

#### Implementation

**Video capture extension.** Video capture extension uses the *MediaStream* Recording API (Mozilla Contributors, 2025) to capture content from a video stream. By default, the device’s primary webcam is used to retrieve the video stream using input parameters, such as resolution and fps, provided during extension declaration. Audio for the stream can be toggled on or off using the declaration parameters. Upon execution, if a proper video input device is not found, the user will be alerted. The extension also handles output files that are recorded from the selected video input device. These files could either contain data for the entire duration of a trial or could be chunked up into segment files using the *timeslice* parameter to improve data transmission on limited network bandwidth. The name for recording files can be assigned independently for each trial using the *filename* parameter in extension properties defined at trial definition. In the case of chunked video files, a timestamp is appended to the filename of each chunk belonging to a trial’s recording. The output files from this extension can either be downloaded locally (default behaviour) or sent to a hosted server using server-side platforms such as JATOS (Lange, Kühn & Filevich, 2015). The extension provides compatibility for JATOS if an instance is detected.

**Video capture setup plugin.** Video capture setup plugin enumerates through available media devices to find video input devices (webcams) and stores their device IDs and names. The list of devices is presented as a drop-down alongside a live preview of the incoming video stream from the default selected device. If a device with no valid input is selected, the live preview is replaced with an error message suggesting that the participant choose a different device from the drop-down menu. The live preview is automatically updated when switching between devices in the drop-down menu. The instructions for the user are displayed on the top of the page and can be customized using the *instruction* parameter of the plugin. Upon proceeding to the next node in the timeline, the experiment will lock the selected device and use its stream to collect video data for any subsequent trials that use the video capture extension, in the background.

### Audiovisual response plugin

There exist many web-based tools for the presentation of audio and visual stimuli independently; however, combining the two kinds of stimuli is not always straightforward, given the differences in web APIs for handling each data type. Nonetheless, presenting combined audiovisual stimuli is a common practice in behavioural experiments. The audiovisual response plugin is a solution for displaying static or animated (GIFs) images with audio simultaneously, while allowing precise timing control and registering responses from the user. The plugin parameters customize trial duration, visual stimulus display size, time delay before which the trial can be pre-empted, whether a response is permitted while audio is playing, and whether a participant’s response, or the end of the audio, will end the trial. Temporal accuracy is an obvious concern with auditory stimuli, therefore, the plugin outputs data fields providing accurate timestamps for stimulus onset and offset. The plugin can also collect user responses in a variety of ways using button(s) and slider inputs, see Fig. 2 for a visual depiction.

This plugin can be used in any experiment that involves the simultaneous presentation of audio and visual stimuli. For example, we are using this plugin in combination with the video capture extension and setup plugin (introduced in the previous section) to run a follow-up eye-tracking study of Fink, Fiehn & Wald-Fuhrmann (2024) where participants explore static art pieces with or without background music, while their webcam data is recorded to track their gaze on the screen. Another straightforward application of this plugin would be to replicate established findings in visual behaviour research, now incorporating auditory elements to examine multimodal interactions.

#### Implementation

Many of the functions in this plugin are heavily dependent on specific parameters, as they change how the trial is set up and how participants can progress. The visual stimulus can be presented either as an HTML <*canvas* > or <*img* > element. Generally, an <*img* > element is more widely supported and responsive, while the <*canvas* > element is more complex, which allows for more spatial and temporal control when presenting. Rendering as a <*canvas* > element can also avoid blank screens appearing when presenting multiple image trials consecutively, reducing eye strain for the participant. On the other hand, if the visual stimulus is an animated GIF, rendering as an <*img* > element is recommended, as <*canvas* > elements are less responsive and only present static images. The *stimulus_height*, *stimulus_width*, and *maintain_aspect_ratio* parameters are used to scale the visual stimuli. User input for this plugin can be collected either with buttons (using the *choices* and *multi_button_response* parameters) or a slider (controlled using slider parameters like *show_slider*, *slider_prompt*, *slider_step*, *etc*.).

The audio file is retrieved and played from the provided path in the plugin parameters. There are a few different ways for the trial to end depending on the parameters chosen. As a result, whether or not response buttons are displayed and how they are used can also vary between trials. The trial can end after a specified duration (using *trial_duration*), after a response (using *response_ends_trial*), or at the end of the audio (using *trial_ends_after_audio*). The plugin also allows adding a time delay before a user can input their response (*button_activate_time*) or specifying if responses should be allowed at all while the audio is playing (*response_allowed_while_playing*). Timestamps for onsets and offsets of audio and visual stimuli are stored in the plugin output using either the more precise *Performance.now()* method or the traditional UNIX epoch styled *Date.now()* method (set with *use_date_now* parameter).

### YouTube response plugin

Video stimuli are a part of many experimental paradigms, which has motivated the development of video-response plugins in jsPsych that display a video stimulus and register user’s responses using a keyboard, button, or slider input. With the proliferation of online content often governed by intellectual property restrictions, a common need of experimenters is to incorporate videos or livestreams from online media sources like YouTube instead of self-hosted files. The YouTube button response plugin expands on the existing video-response plugins by utilizing a YouTube player for displaying both videos and livestreams. This plugin allows playing a public or unlisted YouTube livestream or video by providing the appropriate URL in the plugin parameters. Users can input responses with customisable buttons and/or a slider (demonstrated in Fig. 2). The trial length can be customized independently of video length, and trials may end after the stimulus or wait indefinitely for a participant’s response. Additionally, experimenters can control whether the YouTube player controls (*e.g.*, play/pause) are enabled, if media autoplay is enabled, and whether the video is muted or not. These parameters provide the experimenter with control over how much a user can interact with the livestream or video playback. Experimenters can choose to minimize participant interference, or allow them to adjust the playback depending on the experimental procedure requirements. This plugin also allows adding a tolerance for the amount of time spent in buffering or paused states where the video playback is paused. These states can trigger due to network or user actions. However, unintended triggering of these states can disrupt the planned experimental flow and lead to inefficient use of the participants’ time. Since the experimenter cannot explicitly control playback states in remote settings, the plugin allows setting a timeout parameter after which the trial is terminated, providing a more responsive interface. Note that this plugin loads content from YouTube to the client’s device, during which YouTube (*i.e.,* Google) may receive standard connection information such as participants’ IP addresses and device details. When using in a study, experimenters should carefully consider the privacy implications of this data sharing and appropriately inform the participants while obtaining consent.

This plugin has direct applications for studies exploring user interactions or behaviours with popular online content, such as social media reels or live premieres. In a study conducted by Schlichting (2025), this plugin was used to stream a live concert performance to remote, online participants and compare subjective responses between in-person and remote attendees. In combination with webcam-based eye-tracking (Saxena, Fink & Lange, 2024) and/or heart-rate measurements (Boccignone et al., 2025), this plugin paves the path to investigating behavioural and physiological responses in remote, online studies. Such studies improve ecological validity and can be used to perform systematic comparisons between in-person *vs.* remote settings in lectures, concerts, sport events, *etc*. Thus, combining the YouTube response plugin with the Video capture plugin and extension provides an out-of-box solution to collecting multi-modal data during socially-relevant, naturalistic remote settings.

#### Implementation

YouTube response plugin uses the HTML <*iframe* > element and the *YouTube Player API* (Google Developers, 2025) to embed a YouTube stream in the trial. The source for the YouTube stream can be set using the *stimulus* plugin parameter. The plugin defines event listeners for tracking stream-related events triggered in the YouTube Player. *onPlaybackQualityChange* is triggered when the quality of the video increases or decreases and records the new quality (*e.g.*, hd720, hd1080, *etc*.) alongside a timestamp of when this change took place. Additionally, *onPlayerStateChange* triggers when the video starts playing, buffering, or is paused, and records these events in a similar format. The *onPlayerReady* trigger records the initial playback quality of the video player if everything is initialized properly. The plugin also maintains a *playerInfoStates* array, logging events every 5 seconds by default. This time interval can be changed through the *log_after_every* parameter. This array is meant to assist post-hoc timestamp synchronisation between the different streams of recorded data. The logged-in data depicts the time on the client, the current playback time of the media, whether or not the viewer is watching at the live head of the media, and the total duration of the media. All timestamps can be recorded either using the more precise *Performance.now()* method or the traditional UNIX epoch styled *Date.now()* method with the *use_date_now* parameter. A timeout for buffering and paused states can be set using the *buffering_timeout* parameter. If the player remains in either state beyond this limit, the plugin ends the trial automatically.

The *prompt* parameter can be used to display prompts under the video player. Response buttons and sliders are configurable in the same way as in the audiovisual response plugin. Like its counterpart, this plugin can terminate a trial after a specified duration (using *trial_duration*), or after a user response (using *response_ends_trial*) and it allows adding a time delay before a user can input their response (*button_disable_time*). Finally, the duration for which video is played can be set independently from the trial duration using the *stimulus_duration* parameter, and player behaviour/interaction can be controlled using the *pointer_events*, *mute*, *autoplay*, and *controls* parameters.

### Path animation display plugin

**Figure 3:**
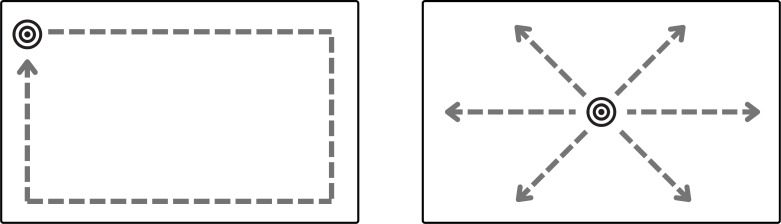
Default paths in **path animation display plugin**–rectangle (left) and line (right). A target appears and waits for a key press. Once pressed, it moves along a path (the outlined paths in the figure are not visible at execution, only the moving target is visible) until it reaches its endpoint or completes the set repetitions. The starting point, movement direction (clockwise or anti-clockwise), target speed, path dimensions, and movement angle for the line path can be configured with plugin parameters. Custom paths can also be provided in the plugin parameters.

In addition to displaying static visual stimuli, experiments often require moving stimuli displayed dynamically at different locations on the screen. These advanced manipulations of positioning and timing are not directly possible with existing image presentation options. The next two plugins aim to provide this functionality. Path animation display plugin animates a visual target in a predefined 2D path. The plugin provides two default paths–a rectangle and a line; see in Fig. 3. Specific properties of the path trajectory such as the starting location, dimensions, pace, line angle, and animation direction (clockwise or anti-clockwise) can be configured with the plugin parameters. In addition to the default trajectories, the plugin can also take a custom animation function to enable user-defined motion. Any image file can be selected as the moving visual target and displayed at a specified size on the screen. The target begins moving after a keypress event from the user and the trial ends once the target finishes moving along its path. To loop the path animation multiple times, the number of repetitions can be set in the plugin parameters at initialisation. The target’s location coordinates at each rendered position can optionally be stored as part of the plugin’s recorded data. Moreover, the mouse pointer can be set to hidden to prevent interference/distractions with the target movement (also the case for the stimulus matrix display plugin).

This plugin can be applied in various contexts. For instance, moving a smooth pursuit target for calibration in eye-tracking studies as presented in Saxena, Lange & Fink (2022). Smooth pursuit calibration improves efficiency of data collection, as it is able to collect many data points in a short period of time without compromising accuracy. We have also applied this plugin for a study designed to evoke back and forth smooth pursuit eye movements, as in Eye Movement Desensitization and Reprocessing (EMDR) therapy where side-to-side eye motion facilitates bilateral brain stimulation.

#### Implementation

The target movement can start either with a valid keypress from the user or automatically with the start of plugin execution. Once the movement starts, the *location_coordinates* function will return the coordinates of the target based on its progress and the chosen value of *path_shape*. The locations can also be fetched from a user-defined path provided with the *custom_path_function* parameter. In the case of a rectangular path, the plugin calculates the coordinates based on the provided dimensions in *path_breadth* and *path_length* parameters. For the line path, the plugin uses *path_length* and *path_slope* to calculate presentation coordinates. The path can be further configured by changing the *starting_location* and *clockwise* parameters. The *stimulus_height* and *stimulus_width* parameters determine how large the target appears to the participant. During animation, the target’s position is continuously updated using new coordinates as the animation progresses, until the progress is complete. The speed of updates is determined by the *animation_duration* parameter provided by the experimenter. If there are still more *repetitions* to be done, the *start_time* variable is reset and the animation repeats until the desired number of repetitions is completed.

### Stimulus matrix display plugin

**Figure 4:**
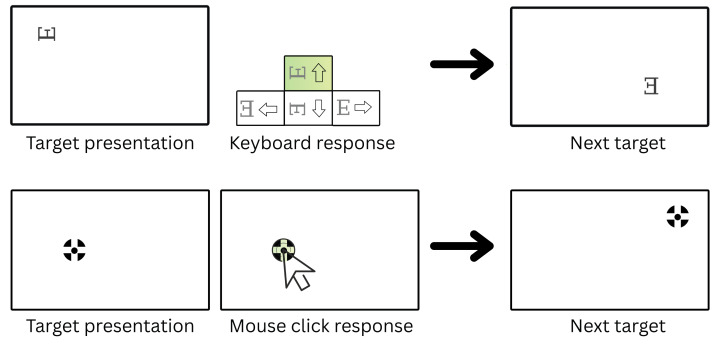
Different configurations possible with the stimulus matrix display plugin for eye-tracking calibration trials. Row 1: A target (character ‘E’ in this case) appears at a random internal grid coordinate, rotated in one of the four possible orientations–0, 90, 180, 270 degrees. The trial waits for the participant’s response and validates the entered response to progress. For *e.g.*, an “up” arrow key response is required to proceed in the shown example. Row 2: The user needs to click on the presented target (image) to proceed to the next one. The grid size, presentation locations, valid responses, and orientation angles can be pre-defined using plugin parameters or randomly selected at execution.

Often experiments require presenting images at discrete locations instead of smooth moving trajectories as presented in the previous plugin. Stimulus matrix display plugin offers a set of such manipulations through easily tunable parameters. The plugin is designed to sequentially present visual stimuli within each cell of a user-defined grid. Experimenters can specify the grid dimensions, as well as the size and duration of each stimulus. The presentation screen is divided into equal-sized cells based on the selected grid layout. More advanced users can also provide a custom drawing function that controls the presentation locations of the stimuli. The visual stimuli can be selected either as an image or a text character and the order of presentation can be randomized or pre-specified in the plugin parameters. Additionally, the presented stimuli can be rotated by either providing a pre-specified rotation array with the desired angle for each presentation location, or randomly selecting a rotation angle for each location. The experimenter can configure trials to advance *via* keyboard input using a choice of valid key responses, mouse clicks on the target, or timed presentation without taking user responses. Figure 4 shows an example of a rotated text stimulus in row 1 and a clickable image stimulus in row 2. When the plugin is not expecting mouse clicks, the mouse pointer can also be disabled from the screen to reduce distractions.

Stimulus matrix display plugin allows presenting visual stimuli in specific zones (grid cells) on the screen as implemented in Saxena, Fink & Lange (2024). Another common application of this procedure is for displaying fixation point-targets in eye-tracking studies. The classic 9-point or 16-point calibration design of presenting a visual target on a 3 × 3 or 4 × 4 grid and taking keyboard or mouse responses from the user can be directly implemented using this plugin. More advanced calibration procedures, such as the fix-point calibration task presented in Saxena, Lange & Fink (2022) that has been validated to provide improved accuracy of gaze predictions and better validation checks in unsupervised online experiments, can also be implemented by choosing appropriate parameters for this plugin. In this case, the fix-point calibration task presented a character (‘E’) target in random locations with random orientations of 0, 90, 180, and 270 degrees. The participant is instructed to direct their gaze to the target and make a response with one of the arrow keys, corresponding to the correct orientation of the ‘E’ (see example in Fig. 4).

#### Implementation

The *target_size* and *canvas_size* parameters can be used to control presentation sizes. The order of grid cells in which the visual stimulus will be presented is randomized by default, unless provided in the plugin parameters. For each grid cell, the *location_cords()* function provides the corresponding centre coordinates depending on the grid dimensions-*-grid_rows* and *grid_cols*. The stimulus is finally drawn with either the default function or a user-provided *stimulus* function. In the default draw function, each target presentation can be rotated at an angle decided by the *trial_rotations* array. By default, the targets will not be rotated. Similar to *trial_locations*, a predefined *trial_rotations* array can be provided in plugin parameters to define the intended target orientation at each presentation location. If a duration for presentation is not provided with *target_duration* parameter, then the plugin waits for the user to either respond by clicking on the target (set with *clickable_targets* parameter) or by a keyboard press. The *choices* parameter can be used to specify valid keyboard responses for each presentation location independently or set to “ALL_KEYS” to allow any key response. The following information is recorded for each target presentation: which response input was provided, reaction time in milliseconds, timestamp when input was provided, and the number of wrong input events before the correct event was made. After a correct input event or target click or end of *target_duration* ms, the *show_next_target* function will present the next target and record data about the index, location, rotation angle, and time of presentation for the next target in the *target_presentation_time* array.

## Using AVOKE

**Figure 5:**
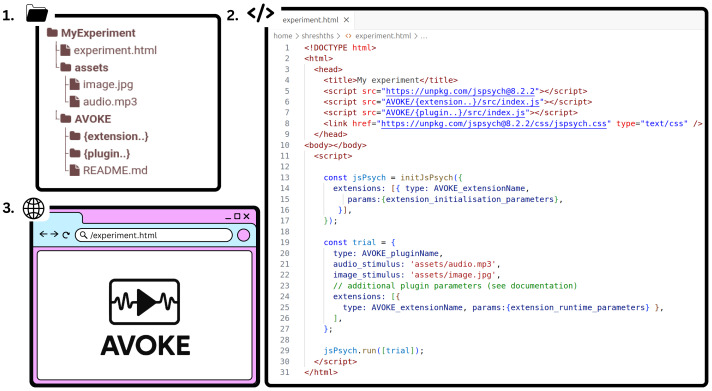
AVOKE workflow and usage. Getting started with AVOKE involves three main steps: (1) set up a directory containing the AVOKE components and create a main HTML file (experiment.html in the above figure); (2) initialize a jsPsych experiment timeline and import the required AVOKE plugins or extensions; and (3) execute or open the HTML file in a web browser to run the AVOKE trials. Example experiments demonstrating these steps are also available in the AVOKE GitHub repository.

AVOKE plugins and extensions can be imported inside any typical jsPsych experiment and included in the experiment timeline. The easiest way is to download or clone the AVOKE repository inside the experiment directory space and then import the respective plugin/extension script into the main experiment HTML document (see steps 1 and 2 in Fig. 5). Once imported, a plugin object needs to be created to include its trial into the experiment timeline. For the video capture extension, an initial call is needed when defining the jsPsych study inside *initJsPsych*, following which every trial that intends to use the extension should also mention the extension name during its creation. Template objects for each component plugin/extension and an exhaustive list of parameters should be referenced from the AVOKE documentation (see ‘Code availability’).

Since AVOKE operates within the existing jsPsych framework and relies on native web technologies, the expected performance when running an experiment is of the same order as that of jsPsych and javascript across major web browsers (see Bridges et al. (2020) for a systematic comparison of jsPsych’s timing accuracy with existing lab-based and online experiment builders). In practice, however, the performance of web-based experiments depends on several system-specific factors, including operating system and web browser version, CPU load at the time of execution, and the type or complexity of the presented media. To obtain a realistic estimate of expected performance, we recommend real-world testing across diverse hardware and software configurations. To facilitate such evaluations, AVOKE provides simulation functions (see ‘Simulation Testing’) that can be used to batch test the operations specific to each plugin released in AVOKE. Live demos of these tests are also available for one-click execution (link provided in ‘Code availability’).

## Simulation Testing

Every plugin and extension introduced in AVOKE is released with corresponding unit tests to validate behaviour across diverse input conditions and to ensure the reliability of core functionalities over repeated trials. While jsPsych supports automated testing using the Jest framework (Meta Open Source, 2024), developing and executing these tests requires familiarity with modern JavaScript tooling and external dependencies—posing a potential barrier for researchers without advanced programming backgrounds. In contrast, AVOKE employs jsPsych’s built-in simulation mode (De Leeuw et al., 2023) to develop its tests. Each test is released with example usage code, enabling straightforward execution and easy customisation. The tests can be directly executed with default configurations for quickly testing cross-device compatibility of plugin functionalities, or adapted easily in simple JavaScript to evaluate custom logic.

In addition to executing pre-defined or customised tests, the simulation functions developed for each plugin and extension also allow generation of simulated behavioural data sets. This simulated data is useful for automated pilot testing, spoof testing, and developing post-processing or analysis scripts for an experiment. The generated datasets can be further tweaked to simulate expected effects and outcomes. Simulation functions developed in AVOKE, therefore, reduce the costs of manual repetitions of trials for testing, debugging, and developing analysis pipelines. The specific simulation functions defined for each of the plugins included in AVOKE are detailed below.

### Video capture extension & setup plugin

**Device enumeration validation.** The test verifies that the plugin accurately enumerates available video input devices by cross-validating the list of detected devices against the results returned by the browser’s MediaDevices API. The tests mock webcam device discovery and validate that the plugin’s *webcam_device_ids* and *webcam_device_names* arrays are properly populated during simulation runs.

### Audiovisual response plugin

**Media resource validation.** The tests systematically verify the loading and accessibility of both audio and visual stimuli. Image load tests confirm successful loading of visual stimuli through JavaScript Image objects, while audio load tests validate audio file accessibility using HTML5 Audio elements. Failed media loading is detected and reported, ensuring stimulus integrity before experimental deployment.

**Aspect ratio preservation testing.** The test validates that visual stimuli maintain their original aspect ratios when scaled according to specified dimensions. The tests calculate expected dimensions based on original image proportions and compare them against plugin-rendered dimensions, ensuring that stimulus presentation remains visually accurate and standardized across different display configurations, if the user chooses to do so.

### Youtube response plugin

**URL validation testing.** The tests systematically verify that stimulus links conform to valid YouTube URL formats using regular expression pattern matching. This validation ensures that both standard YouTube.com and shortened youtu.be URLs are properly recognized, preventing experimental failures due to malformed video links and ensuring cross-platform compatibility across different YouTube URL formats.

**Temporal accuracy validation.** The simulations validate *trial_duration* precision by comparing actual trial durations (calculated from *start_time* and *end_time* timestamps) against expected *trial_duration* parameters. The tests employ a 15-millisecond tolerance threshold to account for browser rendering variability and network latency while ensuring accurate timing control essential for multimedia presentation experiments.

**Player information logging consistency.** The testing suite validates the consistency of YouTube player state logging by examining the playerInfo array against the specified *log_after_every* parameter. The tests verify that logging intervals remain consistent throughout video playback, ensuring reliable data collection for analysing participant engagement patterns and video interaction behaviours.

**Timestamp sequence validation.** The simulations validate *playerTimestamps* data integrity by verifying that all timestamp entries maintain ascending chronological order. This testing ensures that temporal data remains consistent and that video playback timing information can be reliably analysed for behavioural research applications.

### Path animation display plugin

**Spatial boundary validation.** The tests systematically verify that all target presentation locations remain within the defined path boundaries by checking each coordinate pair against the specified *path_length* and *path_breadth* parameters. This boundary testing ensures that animated stimuli do not appear outside the designated experimental space, maintaining spatial validity across different screen resolutions and path configurations.

**Animation duration precision testing.** The simulations validate temporal accuracy by comparing actual animation durations (calculated from *start_time* and *end_time* timestamps) against specified *animation_duration* parameters. The tests employ a 3-millisecond tolerance threshold to account for browser rendering variability while ensuring precise timing control essential for eye-tracking experiments.

**Response key validation.** The testing suite verifies that participant responses fall within the predefined set of allowed choices, ensuring data integrity and preventing invalid key presses from contaminating experimental datasets. This validation is particularly important for experiments requiring specific response mappings.

**Repetition cycle verification.** The tests validate that repetition numbers in the *target_presentation_time* data fall within the expected range (1 to *repetitions_set*), ensuring that the animation cycling behaviour operates correctly across multiple repetitions of the same path trajectory.

**Temporal ratio consistency.** The simulations validate the values of ratios in the presentation timeline, ensuring all temporal ratios remain within the valid range [0, 1]. This testing guarantees that target positions are correctly interpolated along the animation timeline and that temporal spacing remains mathematically valid.

### Stimulus matrix display plugin

**Response key validation.** The tests systematically verify that participant key presses fall within the predefined set of allowed choices, handling both specific key arrays and “ALL_KEYS” configurations. This validation ensures data integrity by detecting invalid responses that could compromise experimental datasets, particularly important for directional response tasks where arrow keys must match stimulus orientations.

**Error accumulation accuracy.** The simulations validate the calculation of total wrong inputs by comparing the aggregate sum of individual response errors against the plugin’s calculated *total_wrong_inputs* parameter. This verification ensures accurate error tracking across multiple stimulus presentations within a single trial, critical for experiments measuring attention lapses or response accuracy patterns.

**Spatial boundary validation.** The tests verify that all stimulus presentation locations remain within the defined canvas boundaries by checking each coordinate pair against the specified *canvas_size* parameters. This boundary testing ensures that stimuli appear within the visible experimental space across different screen resolutions and grid configurations, maintaining spatial validity for attention and visual search experiments.

**Multi-modal stimulus testing.** The testing suite validates plugin behaviour across diverse stimulus types, including text characters, image stimuli (the simulations use Chicago Face Database examples), and clickable targets. Each modality is tested with different grid configurations (3 × 3, 4 × 4, 2 × 2) to ensure consistent spatial distribution and response collection.

**Interaction mode validation.** The simulations test both time-based stimulus presentation (fixed duration trials) and user-interaction-based presentation (clickable targets), ensuring that response collection mechanisms function correctly across different experimental paradigms. This includes validation of null target durations for click-based interactions *versus* fixed timing for passive viewing tasks.

**Rotational stimulus integrity.** The tests validate proper handling of stimulus rotation angles, including cardinal directions (0°, 90°, 180°, 270°) and custom angle sets (0°, 45°, 90°, 135°, 180°, 225°, 270°, 315°), ensuring accurate spatial orientation for directional discrimination tasks.

## Code Availability

All code comprising the AVOKE toolbox is openly available at https://github.com/beatlab-mcmaster/AVOKE under the MIT License. A live demo webpage with example experimental paradigms developed using AVOKE plugins is also available at: https://beatlab-mcmaster.github.io/AVOKE/. Detailed documentation and demo examples for code usage are provided for each plugin to assist new users. Tests and simulation functions discussed in this paper are also included in the code repository. The live demo webpage allows executing each plugin and pre-defined test without any setup.

## Discussion

To summarize, AVOKE provides easy-to-use implementations of complex audio-visual paradigms commonly applied in experimental research. The tools in AVOKE are built with a focus on (i) Standardisation—using native web technologies and the community-driven jsPsych framework to incorporate advanced methods in web experiments, (ii) Accessibility—providing clear documentation for inexperienced users to employ and modify these procedures with little resistance, and (iii) Reliability—providing simulation functions for automated testing and generation of plausible behavioural data. AVOKE aims to contribute to the overarching goal of reducing the gap between remote and laboratory-based experimentation, enabling fast and easy remote data collection from global populations. The current release of the AVOKE toolbox, discussed in this paper, includes five different plugins for customisable audiovisual stimulus presentation + response collection, and an extension that executes in the background of any plugin to record webcam video data, enabling inference of advanced behavioural and physiological measures like gaze, blinks, head motion, and cardiac activity.

While AVOKE makes the process of experiment building (stage 1 in Fig. 1) easier, open, and reproducible, the overall accessibility, scalability, and data privacy when conducting an experiment is a responsibility of the experimenter and the hosting/deployment (stage 2 in Fig. 1) solution used in conjunction. When building the experiment, we encourage users and experimenters to adhere to universal design principles (Story, Mueller & Mace, 1998) and web-content accessibility guidelines—WCAG 2.2 (World Wide Web Consortium, 2024) that improve the accessibility of digital content for people with disabilities (Vereenooghe, 2021). When deciding upon a hosting/deployment solution, experimenters should carefully consider its storage and bandwidth limits to avoid data collection bottlenecks and ensure that their data handling and privacy practices comply with the institutional, funding, and regional regulations (*e.g.*, the European Union’s General Data Protection Regulation–2016). Finally, it is critical that researchers obtain ethical approval from appropriate review boards, prior to data collection, and ensure that participants are clearly informed about the intended use of their data (see Bhutta, 2004 for best practices).

With this release of AVOKE, we seek to advance reproducible, user-friendly, and inclusive web-based research while contributing to the growing open-source ecosystem around jsPsych. Future work will focus on expanding the capabilities of AVOKE through community feedback and collaboration. We encourage new users and readers to try out our demo examples (links available in ‘Code availability’) and look forward to contributions and feedback from more experienced users.

## Additional Information and Declarations

### Data Availability

The following information was supplied regarding data availability: The code is available at Github and Zenodo: - https://github.com/beatlab-mcmaster/avoke
- Shi, J., Saxena, S., & Fink, L. (2025). AVOKE: an open-source web-based experimentation toolbox for evoking audiovisual responses (v1.0.0). Zenodo. https://doi.org/10.5281/zenodo.16581811.
